# Podoconiosis and Soil-Transmitted Helminths (STHs): Double Burden of Neglected Tropical Diseases in Wolaita Zone, Rural Southern Ethiopia

**DOI:** 10.1371/journal.pntd.0002128

**Published:** 2013-03-14

**Authors:** Bineyam Taye, Bereket Alemayehu, Asaye Birhanu, Kassu Desta, Sisay Addisu, Beyene Petros, Gail Davey, Aster Tsegaye

**Affiliations:** 1 School of Medical Laboratory Sciences, Faculty of Medicine, Addis Ababa University, Addis Ababa, Ethiopia; 2 School of Public Health, Faculty of Medicine, Addis Ababa University, Addis Ababa, Ethiopia; 3 Wolaita Sodo University, Wolaita Sodo, Ethiopia; 4 Department of Biochemistry, Faculty of Medicine, Addis Ababa University, Addis Ababa, Ethiopia; 5 Faculty of Biological Sciences, Addis Ababa University, Addis Ababa, Ethiopia; 6 Brighton & Sussex Medical School, Brighton, Sussex, United Kingdom; London School of Hygiene and Tropical Medicine, United Kingdom

## Abstract

**Background:**

Both podoconiosis and soil-transmitted helminth (STH) infections occur among barefoot people in areas of extreme poverty; however, their co-morbidity has not previously been investigated. We explored the overlap of STH infection and podoconiosis in Southern Ethiopia and quantified their separate and combined effects on prevalent anemia and hemoglobin levels in podoconiosis patients and health controls from the same area.

**Methods and Principal Findings:**

A two-part comparative cross-sectional study was conducted in Wolaita zone, southern Ethiopia. Data were collected from adult patients presenting with clinically confirmed podoconiosis, and unmatched adult neighborhood controls living in the same administrative area. Information on demographic and selected lifestyle factors was collected using interviewer-administered questionnaires. Stool samples were collected and examined qualitatively using the modified formalin-ether sedimentation method. Hemoglobin level was determined using two different methods: hemoglobinometer and automated hematology analyzer. A total of 913 study subjects (677 podoconiosis patients and 236 controls) participated. The prevalence of any STH infection was 47.6% among patients and 33.1% among controls (p<0.001). The prevalence of both hookworm and *Trichuris trichiura* infections was significantly higher in podoconiosis patients than in controls (AOR 1.74, 95% CI 1.25 to2.42, AOR 6.53, 95% CI 2.34 to 18.22, respectively). Not wearing shoes and being a farmer remained significant independent predictors of infection with any STH. There was a significant interaction between STH infection and podoconiosis on reduction of hemoglobin level (interaction p value = 0.002).

**Conclusions:**

Prevalence of any STH and hookworm infection was higher among podoconiosis patients than among controls. A significant reduction in hemoglobin level was observed among podoconiosis patients co-infected with hookworm and ‘non-hookworm STH’. Promotion of consistent shoe-wearing practices may have double advantages in controlling both podoconiosis and hookworm infection in the study area.

## Author Summary

Podoconiosis and soil-transmitted helminth infections are neglected tropical diseases occurring among barefoot people in areas of extreme poverty, and both promote poverty through effects on education, economic productivity and disability. In Ethiopia, little research on podoconiosis has been conducted and though social, economic and psychological burdens have been described, no previous study has investigated co-morbidity with other neglected tropical diseases. We therefore aimed to explore the overlap of soil-transmitted helminth infection and podoconiosis in southern Ethiopia by comparing the prevalence of STH infections among podoconiosis patients and healthy controls. We also demonstrate the separate and combined impact of STH infection and podoconiosis on hemoglobin level. We found that the prevalence of any STH and hookworm infection was higher among podoconiosis patients than among controls. A significant reduction in hemoglobin level was observed among podoconiosis patients co-infected with hookworm and ‘non-hookworm STH’. Based on the current findings, integrated control programs that include targeted anthelminthic distribution to control STH among podoconiosis patients, and promotion of consistent shoe-wearing practices are recommended in the study area.

## Introduction

People living in rural areas of low-income countries are commonly affected by more than one of the neglected tropical diseases (NTDs). These conditions share common risk factors including lack of clean water or poor sanitation, and commonly promote poverty through their impact on child health and development and economic productivity [1], [2]. There is increasing global interest in geographical overlap in distribution of NTDs and in interactions in their effects on human health [3]. We describe a study aimed to explore the overlaps and interactions between two NTDs common in highland Ethiopia: podoconiosis and soil transmitted helminth (STH) infection.

Podoconiosis is a geochemical (non-filarial) elephantiasis thought to be caused by the absorption of ultrafine mineral particles from the soil through the skin of the feet [4], [5]. In Ethiopia, 11 million people are at risk through exposure to irritant soil, and an estimated 500,000 to 1 million people are affected nationwide [6]. Despite its public health significance in Ethiopia, there is little current information on podoconiosis distribution, though maps from the 1970s [Price, 1976] suggest that areas in which podoconiosis has been documented may overlap with areas of high STH prevalence [7]. Like STH infection, podoconiosis occurs in barefoot populations in areas of great poverty where subsistence farming is the main occupation [8], [9].

In Ethiopia, the distribution and prevalence of STHs vary from region to region because of variations in environmental, social and geographic factors [10], [11], [12]. ‘This Wormy World’ (http://www.thiswormyworld.org/maps/ethiopia) used data from 269 surveys of STHs in Ethiopia from 1981–2009 to predict countrywide distribution [13]. These predictions indicate that most of highland Ethiopia is ‘likely’ or ‘very likely’ to have STH prevalence exceeding 20%.

Intestinal helminths may cause anemia through reduced food intake, malabsorption and endogenous nutrient loss. The main anemia-causing intestinal helminths are hookworms (*Ancylostoma duodenale*, *Necator americanus*), *Trichuris trichiura* and *Schistosoma*, with hookworms being most common. Hookworms cause chronic intestinal blood loss by attaching to the mucosa of the upper small intestine and ingesting tissue and blood [14], [15]. Podoconiosis is thought to be the result of abnormal inflammatory responses to one or more mineral triggers. Little is known of the mediators involved in this inflammatory process, though transforming growth factor β may play a role [16]. We hypothesized that patients with podoconiosis might manifest an anemia of chronic disease, and that anemia might be more pronounced in the presence of anemia-causing intestinal helminths.

In this study, we aimed to explore the overlap of STH infection and podoconiosis in southern Ethiopia by comparing the prevalence of STH infections in podoconiosis patients and healthy neighborhood controls. We also quantified the separate impact of respective infections on hemoglobin levels, and determined the combined impact of podoconiosis and STH on hemoglobin levels.

## Methods

### Study Setting and Context

A two-part comparative cross-sectional study was conducted in Wolaita zone, southern Ethiopia in January/February 2010 and June 2010 representing dry season and rainy season, respectively, in this part of Ethiopia. These two seasons were chosen to minimize any seasonal effect of malaria on the hematological outcomes measured. Wolaita zone was selected because it is known to be highly endemic for podoconiosis [6], and because a local NGO, the Mossy Foot Treatment and Prevention Association (MFTPA) has its base there. The MFTPA provides treatment to approximately 30,000 podoconiosis patients per year, through a carefully structured system of 15 ‘outreach clinics’. This infrastructure has been utilized successfully for previous research studies, and the investigators have a long history of research collaboration with the MFTPA [4], [6], [8], [9]. The ‘outreach clinics’ at which cases were identified are situated between 1300 and 2050 meters above sea level, altitudes at which lymphatic filariasis would be uncommon. The MFTPA has excellent links with the communities in which the outreach clinics are situated, through ‘Network Groups’ that advocate for and support people with podoconiosis.

### Ethical Approval

Approval for the study was given by the Institutional Review Board (IRB) of Addis Ababa University Medical Faculty. The IRB approved use of oral informed consent documented by a witness after the objectives of the study had been explained. All subjects provided informed consent.

### Study Population and Sampling Procedure

Patients 18 years of age and older, presenting with clinically confirmed podoconiosis, and unmatched adult neighborhood controls living in the same administrative area were included. No children were included in the study, because onset of podoconiosis is uncommon under the age of 10. The sample size was originally calculated to investigate differences in T-cell subsets, and was based on the projected standard deviation, which was expected to be considerably larger in cases than controls, based on national reference standards. New adult podoconiosis patients were selected from those attending outreach clinics for the first time, using outreach clinic registration books as the sampling frame for selection of patients. Neighborhoods controls were identified through the MFTPA Network Groups and outreach clinic staff. They were examined carefully to exclude sub-clinical disease before being recruited. Our group has documented that clinical diagnosis of podoconiosis in this endemic area has high validity [17].

### Measurement and Data Collection

After informed consent was obtained from study participants, information on demographic and selected lifestyle factors was collected by interviewer-administered questionnaires. The questionnaires addressed socio-demographic information such as age, sex, educational status, occupational status, disease stage and shoe wearing habits. Each participant was given a leak-proof plastic container with clear instructions on how to provide a faecal sample. The faecal samples were placed in a plastic container containing 10% formalin and transported for analysis to the School of Clinical Laboratory Sciences, Addis Ababa University. Hemoglobin level was determined using two different methods; hemoglobinometer (HemoCue TM, Angelholm, Sweden) and automated hematology analyzer (Abbott Diagnostics, Abbott Park, IL, USA). Hemoglobinometer measurements were made using a fingerprick sample, whereas automated hematology analysis was performed on 5 ml samples, as described below. The 5 ml sample was taken for more detailed hematology and immunology measurements, which will be reported separately. Both HemoCue and automated hematology analyzer are acceptable methods for measuring hemoglobin level and produce comparable results [18], [19].

#### Formol ether concentration technique

After direct examination of stool for protozoa, formol ether concentration was performed. With an applicator stick, approximately 1 g of the stool sample was emulsified in 4 ml of 10% formol ether contained in a tube. An additional 4 ml of 10% formol ether was added to the tube and homogenized. The emulsified faeces was sieved and collected in a tube. The suspension was transferred to a centrifuge tube into which 4 ml of diethyl ether was added. The tube was stoppered and mixed for 1 minute. The stopper was loosened and the tube centrifuged at 1000 g for 1 minute. After centrifuging, the faecal debris was loosened and decanted along with the ether and formol water leaving the sediment at the bottom of the tube. The bottom of the tube was then tapped to re-suspend and mix the sediment. The sediment was placed on the slide, covered with a cover slip and examined with a microscope. The ova/larvae were identified using Cheesbrough's ‘District Laboratory Practice in Tropical Countries’ [20].

### Hemoglobin Measurement

#### Technique 1: Automated hematology analyzer (Cell Dyn 800)

A five ml whole blood sample was collected into Ethylene diaminetetraacetic acid (EDTA) tubes between 9:00 and 11:00am and analysed on the same day using an automated hematological analyzer (Cell Dyn 800). The analyzer aspirates the blood sample, dilutes and counts leukocytes, erythrocytes and thrombocytes, measures Mean Cell Volume and hemoglobin, and calculates Hematocrit, Mean Cell Hemoglobin and Mean Cell Hemoglobin Concentration and RDW.

#### Technique 2: HemoCue method

Capillary blood samples were collected by fingerprick from the left middle finger, using two of the HemoCue specific cuvettes, after cleaning and massaging the finger to stimulate blood flow. The first and second blood drops were collected and approximately 10 microlitres of blood collected into HemoCue cuvettes, which were then inserted into the meter, where the hemoglobin concentration was displayed in grams per deciliter.

### Quality Assurance

The function of the HemoCue photometer was checked on a daily basis by measuring the control cuvette and a standard of known concentration. Three set controls (Low, Normal and High) were run daily to ensure the function of the Cell Dyn 1800.

### Anaemia Determination

Hemoglobin values were used to assess the status of anemia based on the following WHO cut-off levels: below 11 g/dL for pregnant women; below 12 g/dL for non-pregnant women; and below 13 g/dL for men. Severe anaemia is defined as hemoglobin below 7 g/dL [21].

### Statistical Analysis

Data were coded and entered using EPI Info 2002 (Centre for Disease Control and Prevention Atlanta, GA) and analyzed using SPSS version 15 software (SPSS INC, Chicago, IL, USA). Age was grouped into five categories: 15–24 years, 25–34 years, 35–44 years, 45–54 years and 55 years and older. Stage of disease (for podoconiosis patients) was defined according to the staging system developed and tested in the same study setting in 2007 [22]. Four categories of parasite infection were defined: ‘any STH’ if any geohelminth infection was present; ‘hookworm’ if either *A. duodenale*, or *N. americanus* was present; ‘non-hookworm STH’ if any of *A. lumbricoides*, *T. trichuria* or *S. stercoralis* was identified, but not hookworm; and ‘no STH’ if no STH infection of any kind was present. Binary and multiple logistic regressions were subsequently conducted to determine the correlates of prevalent soil transmitted helminth infections. The independent t test was used to compare the mean difference in hemoglobin level across groups. Interactions between hemoglobin levels and categories of parasite infection were computed using multiple linear regression. P-values of less than 0.05 were taken to be statistically significant.

## Results

### Characteristics of the Study Subjects

A total of 913 study subjects (677 podoconiosis patients and 236 controls) were involved. Just under half were male (46.5% of patients and 49.2% of controls, p = 0.49), and patients were older than controls (mean age 39.9 years vs 35.3 years, p<0.001). The great majority of patients and controls (94.3% and 81.4%, respectively) were either illiterate or had not attended school beyond primary level, and most were farmers or housewives. Participants were asked about their shoe wearing history, and 159 (23.5%) podoconiosis patients and 51 (21.0%) controls said they never wore shoes (p<0.001), whereas 25% of patients and 53.4% of controls said they always wore shoes (Table 1).

**Table 1:** Socio-demographic characteristics of podoconiosis patients and neighborhood controls enrolled between January and June 2010 in Wolaita zone, rural Southern Ethiopia.

Variable	Patients (N = 677)	%	Controls (N = 236)	%	P value^1^
**Sex**					
Male	315	46.5	116	49.2	0.487
Female	362	53.5	120	50.8	
**Age**					
**Mean (SD)**	39.9 (13.0)		35.3 (11.7)		<0.001^2^
15–24	69	10.2	39	16.5	
25–34	142	21.0	66	28.0	<0.001
35–44	224	33.1	87	36.9	
45–54	134	19.8	20	8.5	
55 and above	108	16.0	24	10.2	
**Educational status (n = 443)**					
Illiterate	173	58.1	51	35.2	
Primary school (1–8)	108	36.2	67	46.2	<0.001
Secondary school completed	17	5.7	27	18.6	
**Occupation (n = 343)**					
Farmer	159	53.4	55	37.9	
Housewife	80	26.8	44	30.3	
Merchant	32	10.7	28	19.3	0.002
Civil servant	12	4.0	2	1.4	
Student	8	2.7	11	7.6	
Others	7	2.3	5	3.4	
**Shoe wearing history**					
Always	169	25.0	126	53.4	
Sometimes	349	51.6	59	25.6	<0.001
Never	159	23.5	51	21.0	

1 P value was calculated using Chi-squared test.

2 P value was calculated using t test to compare mean difference.

### Prevalence of STH Infection among Podoconiosis Patients and Controls

The results of the faecal examinations are summarized in Table 2. Infection with any STH was detected in 400 (43.8%) study subjects: 322 (47.6%) of the 677 patients and 78 (33.1%) of the 236 controls. The prevalence of ‘any STH’ infection was significantly higher among podoconiosis patients than among controls (AOR = 1.80, 95% CI 1.31 to 2.47, p<0.001). Hookworm was the predominant intestinal helminth infection, detected in 40.9% of patients and in 27.5% of controls, and *Ascaris lumbricoides* was the second most frequently detected intestinal parasite with prevalence of 14.5% in patients and 9.3% in controls. When considering parasites separately, the prevalences of hookworm and *Trichuris trichiura* infections were significantly higher in podoconiosis patients than in controls (AOR = 1.74, 95% CI 1.25–2.42, AOR = 6.53, 95% CI 2.34–18.22, respectively). However there was no significant difference between patients and controls for any other soil-transmitted helminth (Table 2).

**Table 2:** Prevalent soil transmitted helminth infections in relation to podoconiosis infection versus control status in Wolaita zone, rural Southern Ethiopia.

Soil-transmitted helminth species	Groups		Crude OR (95%CI)	Adjusted OR (95%CI)†
	Patients (N = 677)	Controls (N = 236)		
	N (% )	N (% )		
Hookworm^a^				
Yes	277 (40.9)	65 (27.5)	1.82 (1.31–2.51)	1.74 (1.25–2.42)
No	400 (59.1)	171 (62.5)	1	1
Ascaris lumbricoides^b^				
Yes	98 (14.5)	22 (9.3)	1.42 (0.89–2.27)	1.44 (0.90–2.29)
No	579 (85.5)	214 (90.7)	1	1
Trichuris trichiura^c^				
Yes	65 (9.6)	4 (1.7)	6.16 (2.21–17.0)	6.53 (2.34–18.22)
No	612 (90.4 )	232 (98.3)	1	1
Strongyloides stercoralis^d^				
Yes	11 (1.6)	6 (2.5)	0.63 (0.23–1.73)	0.70 (0.25–1.95)
No	666 (98.4)	230 (97.5)	1	1
Any (STH)¥ ^,^ e				
Yes	322 (47.6)	78 (33.1)	1.83 (1.35–2.50)	1.80 (1.31–2.47)
No	355 (52.4)	158 (66.9)	1	

† Multivariate logistic regression adjusting for age, sex, educational status, occupation.

¥ Definition of ‘Any STH’, ‘Double infection’ and ‘Triple infection’ are given in the statistical analysis section.

a,b,c,d,e, The reference group for each geohelminth infection included the following:

^a,b,c,d.^ For individual species of geohelminths: individuals uninfected by any species *or* those infected with geohelminth species excluding that under investigation (eg. for hookworm the reference category includes individuals free of hook worm and those infected with non hookworm geohelminths).

^e^ For any STH infections: individuals uninfected by either species of geohelminth.

### Univariate and Multivariate Risk Factor Analysis for Soil-transmitted Helminth Infection

The relationship between socio-demographic variables and infection with STH was analyzed using univariate and multivariate logistic regression. Being a patient and being a farmer were both significantly positively associated with infection with ‘any STH’. Reported shoe-wearing was also associated with STH infection. Comparing with ‘always’ wearing shoes, the adjusted OR for ‘sometimes’ wearing shoes was 3.40 (95% CI 1.99–5.80), while that for ‘never’ using shoes was 2.45 (95% CI 1.37–4.38, Table 3).

**Table 3:** Clinical and socio-demographic correlates of any soil transmitted helminth infection in Wolaita zone, Southern Ethiopia.

Variables	*Any Soil-transmitted helminth infection*		Univariate Association	Multivariate Association
	*Yes*	*No*	*OR (95% CI )*	*OR(95%CI)* †
***Podoconiosis Status***				
Patients	322	355	1.83 (1.34–2.50)*	1.81 (1.29–2.54)*
Controls	78	158	1	1
***Sex***				
Male	198	233	1.17 (0.90–1.53)	0.844(0.53–1.33)
Female	202	280	1	1
**Age group**				
15–24	47	61	0.79 (0.47–1.32)	0.96 (0.42–2.21)
25–34	81	127	0.65 (0.42–1.02)	0.70 (0.36–1.35)
35–44	136	175	0.80 (0.53–1.20)	0.71 (0.38–1.34)
45–54	71	83	0.88 (0.55–1.40)	0.78 (0.39–1.58)
55 and above	65	67	1	
**Educational status (n = 434)**				
Illiterate	115	106	1.19 (0.61–2.31)	1.17 (0.54–2.55)
Primary school (1–8)	79	92	0.94 (0.48–1.85)	0.89 (0.42–1.89)
Secondary school completed	20	22	1	
**Occupation (n = 434)**				
Farmer	133	76	4.03 (1.98–8.20)*	3.88 (1.84–8.59)*
Housewife	45	74	1.40 (0.66–2.96)	1.20 (0.53–2.67)
Merchant	23	40	1.32 (0.57–3.03)	1.43 (0.60–3.38)
Others	13	30	1	1
**Shoe wearing history**				
Never	124	161	1.96 (1.34–2.85)*	2.45 (1.37–4.38)*
Sometimes	214	194	2.81 (1.97–3.99)*	3.40 (1.99–5.80)*
Always	62	158	1	1
**Stage of disease** ** **(n = 677)**				
Stage III	30	40	0.71 (0.42–1.19)	1.05 (0.50–2.20)
Stage II	100	132	0.72 (0.51–1.04	1.26 (0.65–2.75)
Stage I	192	183	1	

† Multivariate logistic regression adjusting for clinical status, sex, age, education, occupation, shoe wearing habit and stage of disease.

* Statistically significant (P<0.05).

** Data on stage of disease were available only for podoconiosis patients.

### Anemia among Patients and Controls

**Figure 1:**
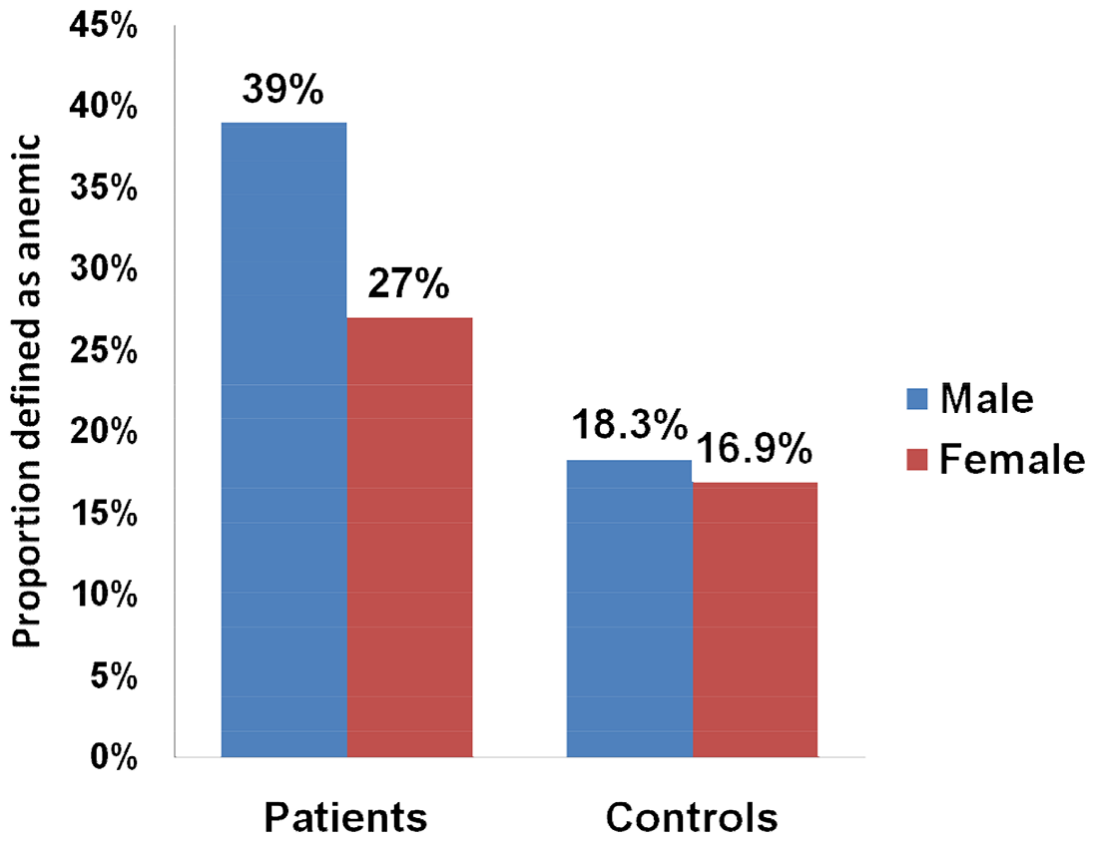
Prevalence of anemia among podoconiosis patients and controls stratified by sex. The prevalence of anemia was significantly higher among male and female podoconiosis patients than among controls.

Mean (SD) hemoglobin level was 13.7 (2.29) g/dl among patients and 14.7 (2.09) g/dl among controls (p<0.001). Hemoglobin level was 13.5 g/dl and 13.9 g/dl (p<0.001) among patients with and without STH infection, respectively, and 14.6 g/dl and 14.9 g/dl among controls with and without STH infection, respectively. Anemia was present in 123 (39.0%) and 100 (27.7%) male and female patients and in 16 (13.8%) and 20 (16.9%) male and female controls, respectively. The prevalence of anemia was significantly higher among male and female podoconiosis patients than among controls (Table 4, Figure 1).

**Table 4:** Hemoglobin level in relation to STH in podoconiosis patients and controls from Wolaita zone, Southern Ethiopia.

STH infection status	Patients	Controls	Crude Association*	Adjusted Association†
	Hemoglobin level	Hemoglobin level	Mean Difference	Mean Difference
	Mean (SD)	Mean (SD)	95%CI	95%CI
All participants	13.7 (2.29)	14.7 (2.04)	0.58–1.24	−1.60 to −0.89
No STH	13.9 (2.15)	14.9 (2.08)	0.36–1.14	−1.39 to −0.54
Any STH	13.5 (2.42)	14.6 (2.09)	0.48–1.66	−2.28 to −1.03
Hookworm	13.5 (2.53)	14.5 (1.77)	0.36–1.67	−2.41 to −1.01

* 95%CI calculated using a two-sample t-test showing the mean difference.

† Adjusted for age, sex, occupation and educational status using multivariate linear regression with hemoglobin as outcome.

A multivariate linear regression model was made to assess the relationship between STH infections and hemoglobin after adjusting for confounding covariates. Among podoconiosis patients, hemoglobin level was inversely related to presence of any STH infection and with hookworm infection (β = −0.59, 95% CI −0.94 to −0.25, and β = −0.56, 95% CI −0.92 to −0.21, respectively). However, no statistically significant association between hemoglobin value and infection with ‘any STH’ was observed among controls (Table 5).

**Table 5:** Soil transmitted helminth infections in relation to hemoglobin levels in podoconiosis patients and controls from Wolaita zone, Southern Ethiopia.

STH infection status	Podoconiosis patients	Controls
	Mean difference (95%CI)†	Mean difference (95%CI)†
Any vs No STH	−0.59 (−0.94 to −0.25)	−0.01 (−0.60 to 0.44)
Any hookworm vs No hookworm	−0.56 (−0.92 to −0.21)	−0.02 (−0.70 to 0.45)
Any vs No *Ascaris lumbricoides*	−0.37(−0.86 to 0.11)	0.02 (−0.67 to 1.01)
Any vs No *Trichuris trichiura*	−0.57 (−1.14 to 0.02 )	0.02 (−1.65 to 2.34)
Any vs No *Strongyloides stercoralis*	0.04 (−0.49 to 2.21)	0.13 (−0.16 to 3.19)

† Estimated from multivariate linear regression models with hemoglobin as outcome and adjustment for age, sex, occupation and educational status.

In the final analysis, the effects of helminth infection in three categories (‘any STH’, ‘hookworm’, and ‘non-hookworm STH’) on hemoglobin level were explored only in patients with podoconiosis. Multiple linear regression analysis with adjustment for age, sex, occupation, educational status showed that ‘any STH’ and ‘hookworm’ infections were both associated with lower hemoglobin levels (−1.07 g/dl, 95% CI: −1.62 to −0.52, and −1.02 g/dl, 95% CI −1.62 to −0.41, respectively, with p-values of 0.001 for each of these interactions). When combined effects were assessed, even greater differences in hemoglobin were observed than with single infections (−1.54 g/dl, 95% CI −2.89 to −0.19 for podoconiosis*non-hookworm STH*hookworm, with interaction p value of 0.002, Table 6).

**Table 6:** Effect of STH and hookworm infections on hemoglobin levels in podoconiosis patients using multivariate regression.

Variable	Beta β**	95%CI	P value *
Podoconiosis * **‘any STH’** †	−1.07	−1.62 to −0.52	0.01
Podoconiosis* **‘hookworm’** ††	−1.02	−1.62 to −0.41	0.01
Podoconiosis * **‘non-hookworm STH’** * **‘hookworm’** †††	−1.54	−2.89 to −0.19	0.02

* Interaction term.

** Adjusted for age, sex, occupation and educational status.

The *reference* group for different categories of geohelminth infections was defined to include the following:

† For any STH infections: Podoconiosis patients uninfected by any species of geohelminth.

†† For hookworm infection: Podoconiosis patients uninfected by any species of geohelminth, and patients infected with non hookworm geohelminths.

††† For non-hookworm STH and hookworm: Podoconiosis patients uninfected by any species of geohelminth, and patients infected with only one species.

## Discussion

This study is the first, to our knowledge, to compare the prevalence of STH infection among people with podoconiosis with that in healthy controls. We found a significantly higher prevalence of STH infections among podoconiosis patients than controls. The overall prevalence of STH infection exceeded that of several other studies in otherwise healthy adult populations in Ethiopia [11], [12], [23]. However, our study showed similar prevalence of STH infection as other studies investigating STH co-infection in patients with HIV and TB in Hawassa (southern Ethiopia), Jimma (south-western Ethiopia) and north Gonder (northern Ethiopia) [24], [25], [26]. Higher prevalence of STH in patients than healthy controls in the latter two studies was related to disease progression and immune responses, suggesting that investigation of the effects of the STH infections on disease progression and immune response in podoconiosis would be useful.

Our study, however, does have limitations. We did not do quantitative egg estimation to measure the intensity of infections because of the lack of adequate laboratory facilities in this very remote rural setting. We cannot therefore provide information on infection intensity.

The results presented here demonstrate a significantly higher prevalence of hookworm among podoconiosis patients than controls, and adjustment suggested that this difference was due to the higher proportion of study subjects not consistently using protective footwear. Hookworm infection was the most common STH infection among controls, consistent with other adult population studies [12], [27]. Although associations between reported shoe use and hookworm infection are inconsistent, Smillie & Augustine [28] report extent of footwear use to be a major factor influencing hookworm transmission, and a recent study in Thailand indicated use of footwear to be the dominant factor in protection against hookworm infection [29]. We assessed the use of footwear by asking participants directly and grading their responses on a simple subjective scale as ‘never’, ‘sometimes’ or ‘always’. We found significantly higher risk of hookworm infection among individuals who ‘never’ or ‘sometimes’ wore shoes than among those that ‘always’ did. Risk of hookworm infection was also non-significantly higher among those who ‘sometimes’ wore shoes than those who ‘never’ did. There are several possible explanations for this – first, that because shoes are considered a valuable asset in rural Ethiopia, they may be removed when working in the fields, a prime time for STH exposure. Secondly, occasional wearing of shoes may have the effect of softening the skin of the feet and permitting easier penetration by third stage larvae. Quantification of shoe wearing in relation to village activities by direct observation (as has been done successfully in relation to water-contact and schistosomiasis [30]), would enable more thorough exploration of the relationship between shoe wearing, STH infection and podoconiosis occurrence. Prevalence of infection with *A. lumbricoides* was not significantly different between podoconiosis patients and controls, presumably because *A. lumbricoides* is transmitted through eating food without washing hands [31], [32]. Though we did not measure this behavior, we assume the probability of exposure by this route is the same for both groups.

Multiple STH infections were more likely among podoconiosis patients than controls. A study conducted in Iran showed that multiple parasitic infections were more frequent in immune-compromised patients than controls, indicating that reduced immunity facilitated establishment of STH infection [33]. Again, future studies of immune status in podoconiosis would be useful in exploring this relationship further.

In the present study we have documented reduced hemoglobin level among podoconiosis patients compared to controls, the difference being significant in each STH infection category. Anemia in podoconiosis patients in the absence of STH infection may reflect the chronic inflammatory process thought to be associated with disease progression in podoconiosis patients [4], or may reflect the marginalisation and undernutrition that are consequences of disease for many patients. Hemoglobin levels were lower in podoconiosis patients infected with any STH than patients without parasitic infection, probably reflecting direct blood loss (through ingestion and mechanical damage of the mucosa) and indirect blood loss by affecting the supply of nutrients necessary for erythropoiesis [34], [35], [36]. Analyses of the separate and combined effects of each STH and podoconiosis on hemoglobin levels suggests that among STH infections, hookworm plays the most important role in influencing hemoglobin levels. However, in terms of magnitude, the effect of podoconiosis on hemoglobin level appears more pronounced than that of hookworm. STH infections appeared to exert an interactive effect on anemia in podoconiosis patients, which is consistent with other studies reporting lower mean hemoglobin among people co-infected with hookworm and trichuris relative to people without infection or with single infection [37].

We conclude that STH infections occur more commonly among podoconiosis patients than healthy controls, suggesting that targeted anthelminthic distribution to control STH among adult podoconiosis patients might be considered in addition to school-based distribution. Further research into the relationship between hookworm infection and shoe use, using more objective measures of shoe use, is urgently needed. This would generate clear evidence for or against shoe wearing in prevention of hookworm infection. If shoe use is found to be protective, integrated prevention of STH and podoconiosis through consistent shoe use must be considered.
